# Comparison of DNA Extraction Methods in Analysis of Salivary Bacterial Communities

**DOI:** 10.1371/journal.pone.0067699

**Published:** 2013-07-03

**Authors:** Vladimir Lazarevic, Nadia Gaïa, Myriam Girard, Patrice François, Jacques Schrenzel

**Affiliations:** Genomic Research Laboratory, Division of Infectious Diseases, Geneva University Hospitals, Geneva, Switzerland; Cairo University, Egypt

## Abstract

Culture-independent high-throughput sequencing-based methods are widely used to study bacterial communities. Although these approaches are superior to traditional culture-based methods, they introduce bias at the experimental and bioinformatics levels. We assessed the diversity of the human salivary microbiome by pyrosequencing of the 16S rDNA V1–3 amplicons using metagenomic DNA extracted by two different protocols: a simple proteinase K digestion without a subsequent DNA clean-up step, and a bead-beating mechanical lysis protocol followed by column DNA purification. A high degree of congruence was found between the two extraction methods, most notably in regard to the microbial community composition. The results showed that for a given bioinformatics pipeline, all the taxa with an average proportion >0.12% in samples processed using one extraction method were also detected in samples extracted using the other method. The same taxa tended to be abundant and frequent for both extraction methods. The relative abundance of sequence reads assigned to the phyla Actinobacteria, Spirochaetes, TM7, Synergistetes, and Tenericutes was significantly higher in the mechanically-treated samples than in the enzymatically-treated samples, whereas the phylum Firmicutes showed the opposite pattern. No significant differences in diversity indices were found between the extraction methods, although the mechanical lysis method revealed higher operational taxonomic unit richness. Differences between the extraction procedures outweighed the variations due to the bioinformatics analysis pipelines used.

## Introduction

Changes in the salivary microbiota are associated with various oral and systemic conditions, including caries, periodontal disease, cancer, arthritis, cardiovascular disease, and obesity [1]–[4]. Studies of salivary bacterial communities initially used culture-based techniques [5], [6]. However, the presence of numerous unculturable bacteria in the mouth, currently estimated to represent about one third of the 600 inventoried species in the curated Human Oral Microbiome Database [7], has necessitated the development of culture-independent approaches. These techniques include DNA-DNA hybridization [8] and high-throughput sequencing (HTS) of 16S rDNA amplicon libraries [1], [9]–[12] or metagenome fragments [13]. The HTS-based methods, now widely used to study bacterial communities, allow the analysis of a small or large number of samples with the desired depth of coverage. Although significantly better than culture-based approaches, the culture-independent methods may introduce bias related to the DNA extraction procedure and the downstream molecular and informatics analyses.

Enzymatic lysis of samples collected using oral swabs [14]–[16] has been used in the study of salivary bacterial communities. This protocol includes treatment with proteinase K, Tween, and EDTA, after which the lysate is used in PCR analyses, either directly [11] or following a clean-up step [13]. In several studies, salivary bacteria were disrupted mechanically using glass, zirconia, or zirconia/silica beads in the presence or absence of phenol, and DNA was purified using different procedures [1], [9], [10], [12].

Here we evaluated two DNA extraction protocols for human saliva samples. DNA extracts obtained by enzymatic or mechanical cell lysis were used to assess bacterial community diversity based on 16S rDNA amplicon pyrosequencing.

## Materials and Methods

### Ethics Statement

This study was approved by the Ethics Committee of the Geneva University Hospitals (09–078). Written informed consent was obtained from the participant.

### Sample Collection

Unstimulated human saliva was obtained as described previously [13] from a 32-year old female non-smoker, without obvious signs of oral disease.

### DNA Extraction

After vigorous mixing by vortex, the saliva sample was divided into six aliquots. Three 100-µL aliquots were mixed with the same volume of 2× lysis buffer (20 mM Tris, 2 mM EDTA pH8, 1% Tween, and 400 µg/mL proteinase K; Fermentas) and then incubated at 55°C for 2.5 h, followed by heating at 95°C for 10 min to inactivate the proteinase K. Three 200-µL saliva sample aliquots were mixed with 700 µL of lysis buffer SL1 (SDS-containing) and 120 µL of Enhancer SX from the NucleoSpin Soil Kit (Macherey-Nagel). The mixture was shaken in a NucleoSpin Bead Tube for 2 min at maximum speed on a Vortex-Genie 2 with a horizontal tube holder (Scientific Industries). From this point, we followed the NucleoSpin Soil Kit protocol (Macherey-Nagel). DNA was eluted in 100 µL of elution buffer SE. DNA extracts from both protocols were stored at −20°C.

### PCR and Sequencing

PCR amplification mixtures included 5 µL of DNA extract, 25 pmol each of forward primer (5′-ctatgcgccttgccagcccgctcag-*ac-*GAGTTTGATCMTGGCTCAG-3′) and a barcoded reverse primer (5′-cgtatcgcctccctcgcgccatcag-NNNNNNNN-*at-*CCGCGGCTGCTGGCAC-3′) in 50 µL Primestar HS Premix (Takara). The composite PCR primers were designed as described previously [11] except that they contained Titanium adaptor sequences (plain lowercase). For each of the six DNA extractions (three enzymatic and three mechanical), two separate PCRs were carried out using reverse primers with a different barcode. All PCRs were performed in duplicate using the following parameters: 30 cycles of 98°C for 10 s, 56°C for 15 s, and 72°C for 1 min. The V1–3 amplicons corresponded to bases 28–514 (excluding primer sequences) in the *Escherichia coli* 16S rRNA gene. Amplicon sizes were checked on a 2100 Bioanalyzer (Agilent). Two replicate PCRs were then pooled and purified using a QIAquick PCR Purification Kit (Qiagen). DNA quantity was assessed using a NanoDrop ND-8000 spectrophotometer (NanoDrop Technologies). Three hundred nanograms of each of the purified samples were then pooled and sequenced from the reverse primer on a Genome Sequencer FLX system with Titanium chemistry (Roche) at LGC Genomics (Berlin, Germany).

### Real-time PCR

To determine the concentration of bacterial DNA, real-time PCR was carried out on an Mx3005P Stratagene cycler using a Brilliant II SYBR Green QPCR Master Mix Kit (Stratagene). Reaction mixtures contained 1 µL of DNA extract, 7.5 pmol each of forward (5′-ACTCCTACGGGAGGCAGCAGT-3′) and reverse (5′-ATTACCGCGGCTGCTGGC-3′) HPLC-purified primers [17] and 0.75 µL of 1/250-diluted reference dye, in a total volume of 25 µL. The cycling conditions included an initial denaturation of 10 min at 95°C, followed by 40 cycles of 95°C for 30 s and 68°C for 1 min. The reference curve for DNA quantitation was created using known concentrations of *Staphylococcus aureus* strain MW2 genomic DNA.

To determine the concentration of human DNA, qPCR was conducted on an Mx3005P Stratagene cycler using ABsolute QPCR Mix (ABgene) and a TaqMan β-actin Control Reagents kit (PE Applied Biosystems). Reaction mixtures contained 2.5 µL of DNA extract, 9 pmol each of β-actin forward and reverse primers, and 6 pmol of β-actin probe (PE Applied Biosystems), in a total volume of 15 µL. The cycling conditions included an initial denaturation of 15 min at 95°C, followed by 42 cycles of 95°C for 15 s and 60°C for 1 min. The reference curve for DNA quantitation was created using known concentrations of human genomic DNA provided with the kit.

### Bioinformatic Analysis

#### Pipeline 1

The fastq file containing 199,205 row sequence reads was deposited in MG-RAST under accession number 4499916.3. Sequence reads were processed using the Mothur (v 1.28) software package [18]. The reads were removed (Mothur’s command trim.seqs) if they met any of the following criteria: (i) contained ambiguous bases; (ii) contained more than one mismatch in the reverse primer (excluding the barcode sequence for which no mismatches were allowed); (iii) contained homopolymer runs longer than 9 bases; (iv) had a minimum quality score of <35 over a 50-base window; (v) had a sequence length <350 or >600 bases after primer sequence trimming. Sequences were aligned (command align.seq) against the Greengenes [19] reference core alignment set (available in Mothur as core_set_aligned.imputed.fasta) and truncated to include bases corresponding to *E. coli* 16S rRNA gene positions 101–514. Sequences that did not cover this region were removed. The commands pre.cluster (with option -diff = 4) and chimera.uchime (default parameters) were used for denoising [20] and removal of potentially chimeric sequences [21], respectively. Samples were normalized to an equal sampling depth corresponding to the size of the smallest sample (command sub.sample). Taxonomic assignment of individual sequences was based on the Naïve Bayesian method [22] and the reference Greengenes taxonomy database (Mothur’s files gg_99.pds.ng.fasta and gg_99.pds.tax), with a confidence score threshold of 80% (command classify.seqs). Sequences were assigned to representative operational taxonomic units (OTUs) using the furthest neighbor method and a 3% dissimilarity cut-off (command dist.seqs and cluster). The consensus taxonomy for the majority of sequences within an OTU was determined using the command classify.otu. A multiple alignment of representative OTUs was imported into FastTree [23] to construct a tree that was then used as the input file for the Fast UniFrac web interface [24].

For Pipelines 2–5, only differences with respect to Pipeline 1 are indicated.

#### Pipeline 2

In this pipeline, potential chimeras were removed using Chimera Slayer (command chimera.slayer) with default parameters [25].

#### Pipeline 3

Instead of chimera removal using UCHIME, sequences were compared with the Greengenes reference database pre-clustered at 97% identity (Greengenes file gg_97_otus_4feb2011.fasta) [19] using BLASTN (-evalue 1e-050 -perc_identity 97 -max_target_seqs 1) [26]. Minimum query alignment coverage was set to 90%. Sequences with no hits were discarded.

#### Pipeline 4

OTU clustering at 97% sequence identity was performed using the CD-HIT-EST web interface [27] (algorithm parameters: G = yes, g = yes, b = 10, al = 0.99, AL = unlimited, as = 0.99, AS = unlimited, s = 0.99, S = unlimited).

#### Pipeline 5

RDP reference database (Mothur’s files trainset9_032012.rdp.fasta and trainset9_032012.rdp.tax) [28] was used for taxonomic assignments.

#### Pipeline 6

Sequences were pre-processed as described in Pipeline 1 (steps i–v), except that the minimum sequence length was set at 200 bases, and residual sequences of the forward PCR primer were removed. Each sequence was mapped to a Greengenes reference OTU by BLASTN, as described in Pipeline 3. Downstream analyses were carried out using the matching full-length 16S rRNA gene sequences and their taxonomic information. The pre-constructed Greengenes reference OTU tree (Greengenes file gg_97_otus_4feb2011.tre) was used as the input file for the Fast UniFrac web interface.

### Clustering of Bacterial Communities

Bacterial community comparisons were carried out with (i) UniFrac, which exploits different degrees of similarity between 16S sequences, and (ii) Bray-Curtis similarity [29], which is a non-phylogenetic metric. Principal coordinates analysis (PCoA) of Bray-Curtis similarities, was performed in PRIMER-E (Primer-E Ltd., Plymouth, UK). Ecological indices were calculated from OTU relative abundance in PRIMER-E.

### Statistical Analysis

Indicator taxa were determined by indicator species analysis [30] (for each taxonomic level individually) using a Monte Carlo test of significance with 1,000 iterations. The *P*-values were adjusted for multiple testing according to Benjamini and Hochberg [31].

Error values are given as standard deviation unless otherwise indicated. Permanova (Primer-E Ltd., Plymouth, UK) and Mann-Whitney U tests were used to assess statistical significance.

## Results

### Sample Preparation and Bioinformatics Analysis Pipelines

DNA was extracted in triplicate from a saliva sample using either a mechanical or enzymatic lysis procedure. Real-time PCR revealed a 5.3-fold and 2.5-fold higher yield of bacterial and human DNA, respectively, using enzymatic lysis compared with the mechanical lysis procedure (Table S1). We generated V1–3 16S rDNA amplicon libraries using the same volume of each DNA extract, which corresponded to 0.6–1.3 and 2.3–2.4 ng of bacterial DNA for the mechanical and enzymatic lysis procedures, respectively.

A total of 199,205 raw sequence reads were generated by pyrosequencing of the amplicon libraries. Following quality control, the sequences corresponding to positions 101–514 in the reference *E*. *coli* 16S rRNA gene were extracted and processed by bioinformatics Pipelines 1–5, which use different tools for chimera removal, OTU clustering, or taxonomy assignments. In Pipeline 6, the quality-filtered sequences with a length ≥200 bases were retained and a reference-based OTU-picking was performed. The number of sequence reads obtained after different preprocessing steps (i.e. DNA quality check, chimera removal, and normalization steps) and the number of OTUs determined across different pipelines are presented in Table 1.

**Table 1:** Number of sequence reads, number of OTUs, and ecological indices in enzymatically- and mechanically-processed samples using different bioinformatic analysis pipelines.

Pipeline	1/5	2	3	4	6
**# of reads after the first quality filter**	80,288	80,288	80,288	80,288	134,928
**# of reads after chimera removal**	76,767	78,697	74,182	76,767	123,508
**# of reads after chimera removal in M**	38,038	38,679	36,761	38,038	61,639
**# of reads after chimera removal in E**	38,729	40,018	37,421	38,729	61,869
**Average % of reads identified as chimeras**	4.0±2.2	1.8±1.4	7.2±2.3	4.0±2.2	8.0±2.3
**Average % of reads identified as chimeras in M**	**2.6±0.8**	0.9±0.3	5.8±0.8	**2.6±0.8**	6.8±0.8
**Average % of reads identified as chimeras in E**	**5.4±2.2 (P = 0.041)**	2.6±1.5	8.6±2.5	**5.4±2.2 (P = 0.041)**	9.2±2.8
**# of reads normalized to (per sample)**	43,524 (3,627)	44,340 (3,695)	41,988 (3,499)	43,524 (3,627)	69,552 (5,796)
**# total of OTUs**	480	630	356	352	326
**AVG # of OTUs in M**	214±18	227±20	177±8	163±13	**210±9**
**AVG # of OTUs in E**	195±19	218±34	167±12	146±15	**184±6 (P = 0.005)**
**AVG Chao 1 in M**	313±30	357±47	255±24	227±25	**250±18**
**AVD Chao 1 in E**	281±43	366±72	251±27	200±39	**219±15 (P = 0.041)**
**% of OTUs shared among M**	71.7±4.4	69±4.5	**74.2±2.8**	75.5±4.6	83.2±3.6
**% of OTUs shared among E**	70.5±5.5	65.5±7.8	**72±4.1 (P = 0.026)**	73.4±6	83±2.6
**% of OTUs shared between M and E**	67.2±5.6	64±6.4	69.1±4	70.6±6.3	79.8±6.1
**Average Spearmans’s rho among M based on OUT** **abundance/** ***P*** ** value** *	0.670/ P<10^−52^	**0.663/ P<10** ^−**66**^	0.693/ P<10^−45^	0.718/ P<10^−48^	0.814/ P<10^−62^
**Average Spearmans’s rho among E based on OUT** **abundance/** ***P*** ** value** *	0.685/ P<10^−57^	**0.631/ P<10** ^−**55**^ ** (P = 0.029)**	0.692/ P<10^−41^	0.710/ P<10^−41^	0.824/ P<10^−71^
**Average Spearmans’s rho between E and M based on OTU abundance/** ***P*** ** value** *	0.605/ P<10^−37^	0.587/ P<10^−41^	0.601/ P<10^−25^	0.639/ P<10^−33^	0.722/ P<10^−41^
**Average Spearmans’s rho between E and M based on OTU prevalence/** ***P*** ** value** *	0.629/ P<10^−53^	0.529/P<10^−45^	0.656/P<10^−44^	0.649/P<10^−42^	0.750/P<10^−66^
**Shannon diversity index [H’(loge)] in M**	3.35±0.15	3.41±0.17	3.23±0.13	3.08±0.16	3.60±0.08
**Shannon diversity index [H’(loge)] in E**	3.45±0.15	3.52±0.18	3.30±0.13	3.11±0.15	3.54±0.09
**Simpson diversity index [1-λ] in M**	0.893±0.020	0.895±0.022	0.886±0.019	0.874±0.022	0.934±0.010
**Simpson diversity index [1-λ] in E**	0.908±0.012	0.911±0.013	0.901±0.012	0.897±0.013	0.934±0.007

E, enzymatic lysis; M, mechanical lysis.

When a significant difference (Mann-Whitney U test *P*<0.05) was found between mechanical and enzymatic lysis, the corresponding values are given in bold and *P* value is indicated in parentheses.

* The highest *P* value for any pairwise comparison.

### Taxonomic Assignments

**Figure 1:**
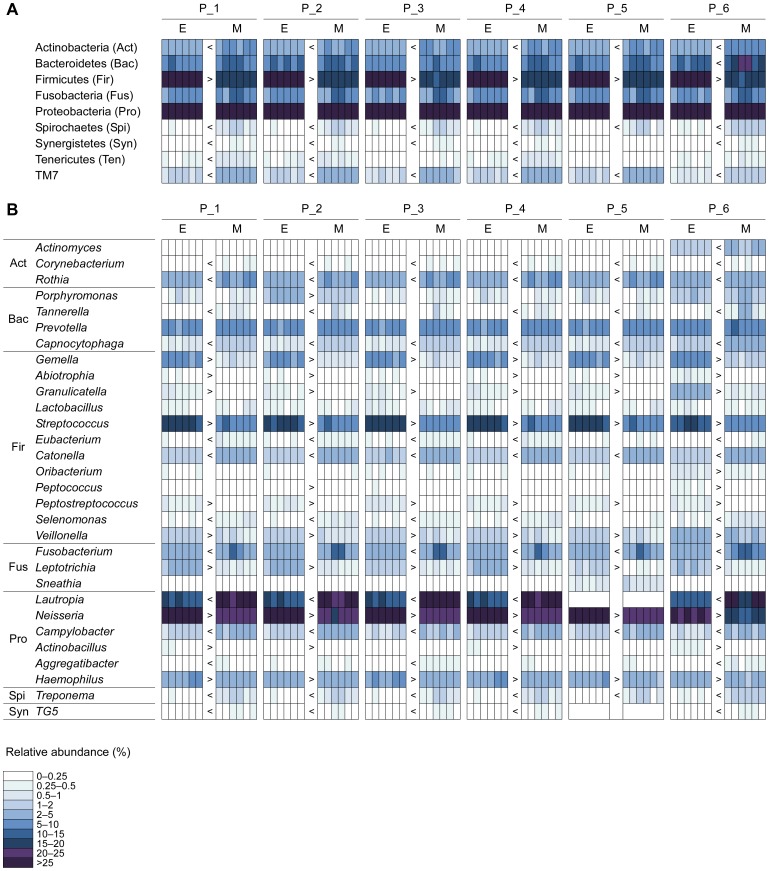
Microbial community profiles for saliva samples at the phylum (A) and genus (B) level. Only taxa found at an average frequency >0.25% by at least one extraction method in at least one analysis pipeline are presented. The indicator values >0.5, determined by indicator species analysis, associated with the Benjamini-Hochberg corrected *P* values<0.05, were used to define indicators. Symbols “>” and “<” correspond to such indicator taxa and denote increasing and decreasing trends in the relative abundance for enzymatic vs. mechanical lysis. Blank cells without borders correspond to taxa names absent from a given taxonomy. E, enzymatic lysis; M, mechanical lysis; P_1–P_6, bioinformatics pipelines 1–6.

In accordance with previous studies [32], seven phyla (Proteobacteria, Firmicutes, Bacteroidetes, Fusobacteria, Actinobacteria, TM7, and Spirochaetes) dominated samples regardless of the lysis method and analysis pipeline used. The phyla Synergistetes, Tenericutes, and SR1 had a relative abundance <1% in all samples (Figure 1 and Table S2).

### Abundance of Taxa

**Figure 2:**
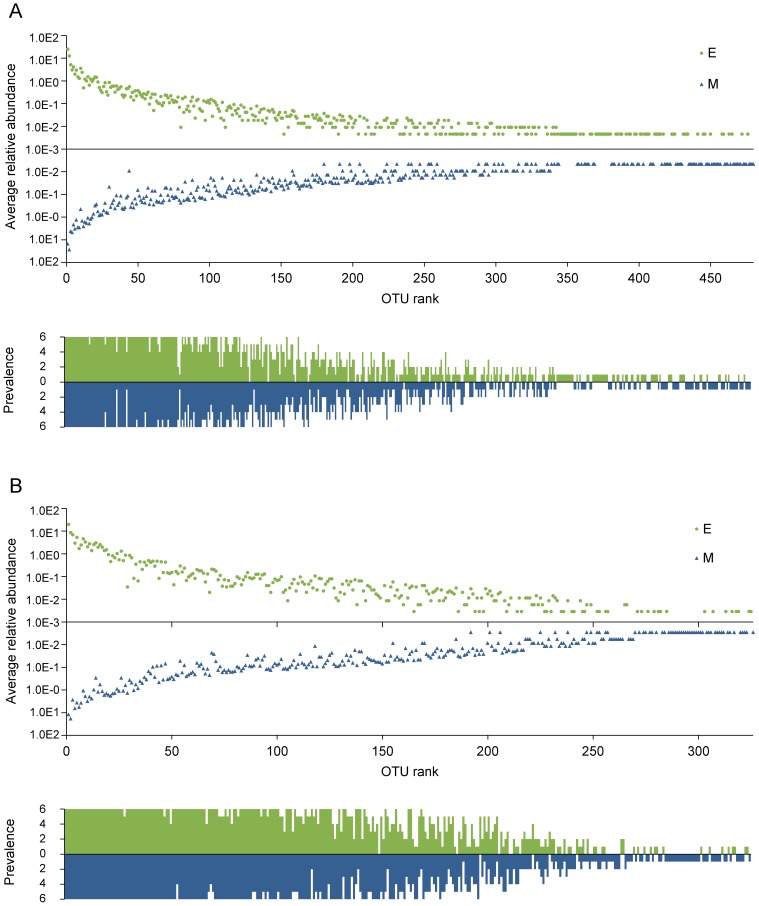
Average relative abundance and prevalence of OTUs determined using Pipelines 1 (A) and 6 (B). The top panel (of each figure) shows the relative abundance of each OTU averaged for the six samples processed with the same extraction method. Individual OTUs are ranked on the x-axis according to their average relative abundance in all (12) samples from high (left) to low (right). E, enzymatic lysis; M, mechanical lysis. Bottom panel indicates the number of samples (0–6) in which the corresponding OTU was found (prevalence). Green bars, enzymatic lysis; blue bars, mechanical lysis.

Assessment of the salivary samples using two extraction procedures revealed a high degree of congruence in terms of the composition and abundance of species in the microbiota. From the phylum level down to the OTU level, all of the taxa with an average proportion >0.12% in samples processed using one extraction method were also detected with the other method, when applying the same bioinformatics analysis pipeline (Figure 1). The most frequent taxa tended to be the most abundant, and the same taxa tended to be abundant and frequent for both extraction methods. These trends, at the OTU level, are exemplified for Pipelines 1 and 6 in Figure 2. The correlation between the extraction methods in terms of OTU abundance and prevalence reached the highest values in Pipeline 6 (Table 1).

Only very low-abundance (average proportion <0.08%) OTUs were present in all six samples derived from one extraction method but absent in all samples obtained with the other method. Two OTUs assigned to the genus *Treponema* (Spirochaetes), along with an OTU belonging to the phylum TM7, were confined to the bead-beating method, whereas an OTU from the genus *Haemophilus* was identified only in samples lysed by enzymatic treatment (Table S2).

Indicator species analysis revealed significant differences in the relative abundance of most major phyla that was related to the extraction procedure (Figure 1). In samples processed by enzymatic lysis, the relative abundance of sequence reads assigned to the phylum Firmicutes was significantly higher than in the mechanically-treated samples, whereas the phyla Actinobacteria, Spirochaetes, and TM7 showed the opposite pattern across all pipelines. The phyla Synergistetes and Tenericutes also had significantly higher relative abundance in mechanically-processed samples, except in Pipeline 5, in which members of this phylum were not identified.

As shown previously [33], different members of the same taxon may present opposite directions of change in relative abundance (increase or decrease) when comparing one extraction method to the other. For instance, the relative abundance of the orders Burkholderiales and Neisseriales, both belonging to the class Betaproteobacteria, were higher and lower, respectively, in mechanically-lysed samples (Figure S1 and Table S2). Similarly, one *Streptococcus* OTU was present at a significantly higher proportion in samples treated mechanically, whereas the abundance of other OTUs from the same genus was significantly greater in enzymatically-lysed samples.

### Clustering of Bacterial Communities

**Figure 3:**
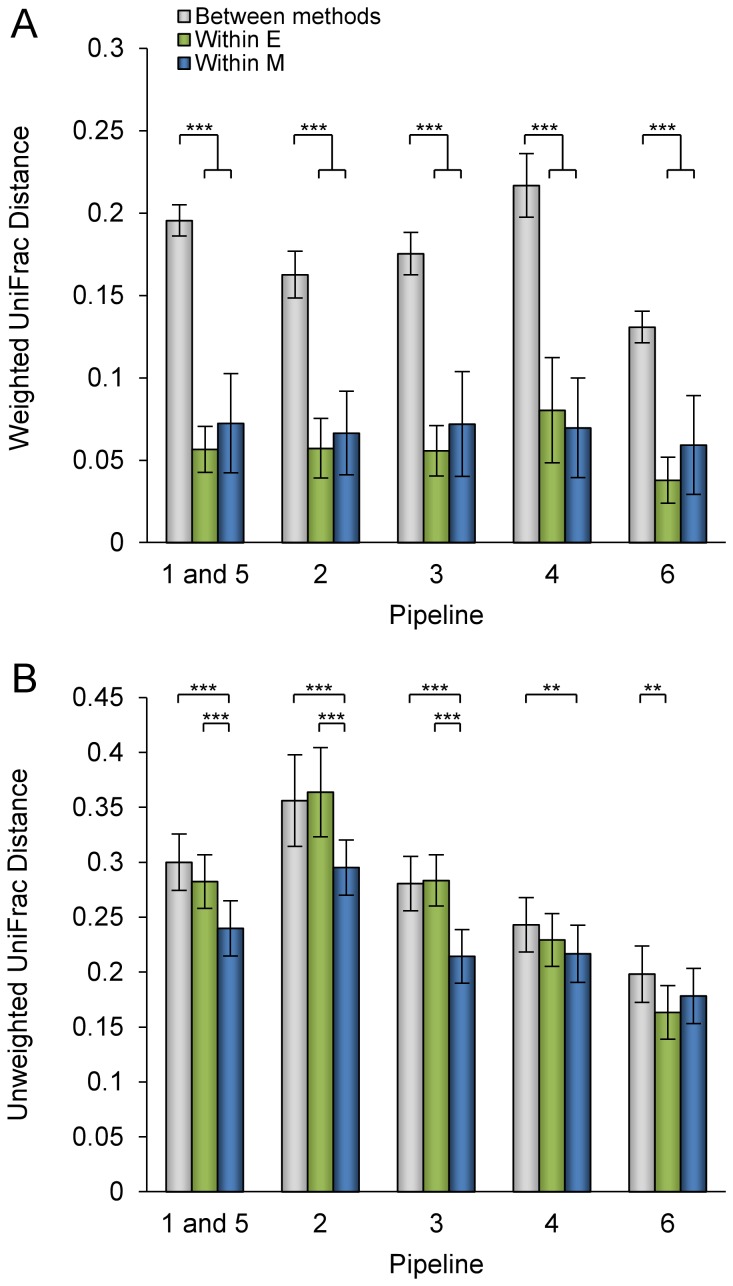
Average between-method and within-method UniFrac distances. (A) Weighted UniFrac. (B) Unweighted UniFrac. Error bars correspond to standard error. The statistical analysis method used was a Mann-Whitney U test. *, P<0.05; **, P<0.01; ***, P<0.001.

Similarities between bacterial communities in terms of their phylogeny were assessed by measuring UniFrac distance [34]. This metric takes into account the relative abundance of OTUs (weighted data) or their presence/absence (unweighted data). The between-method weighted UniFrac distance was significantly higher (*P*<10^−15^) than the within-method distance across all pipelines (Figure 3A). The within-method distances for the mechanical and enzymatic lysis methods were not significantly different.

Comparisons of bacterial community membership using unweighted UniFrac (Figure 3B) showed that intra-method distances were significantly smaller for the mechanically-treated samples than for the enzymatically-treated samples in the pipelines in which OTUs were clustered using Mothur (Pipelines 1/5, 2 and 3). In these pipelines, as well as in Pipeline 4, UniFrac distances between the mechanically-lysed samples were also smaller than inter-method distances. In contrast, in Pipeline 6, a statistically significant decrease was observed when unweighted UniFrac distances among enzymatically-processed samples were compared with inter-method distances.

**Figure 4:**
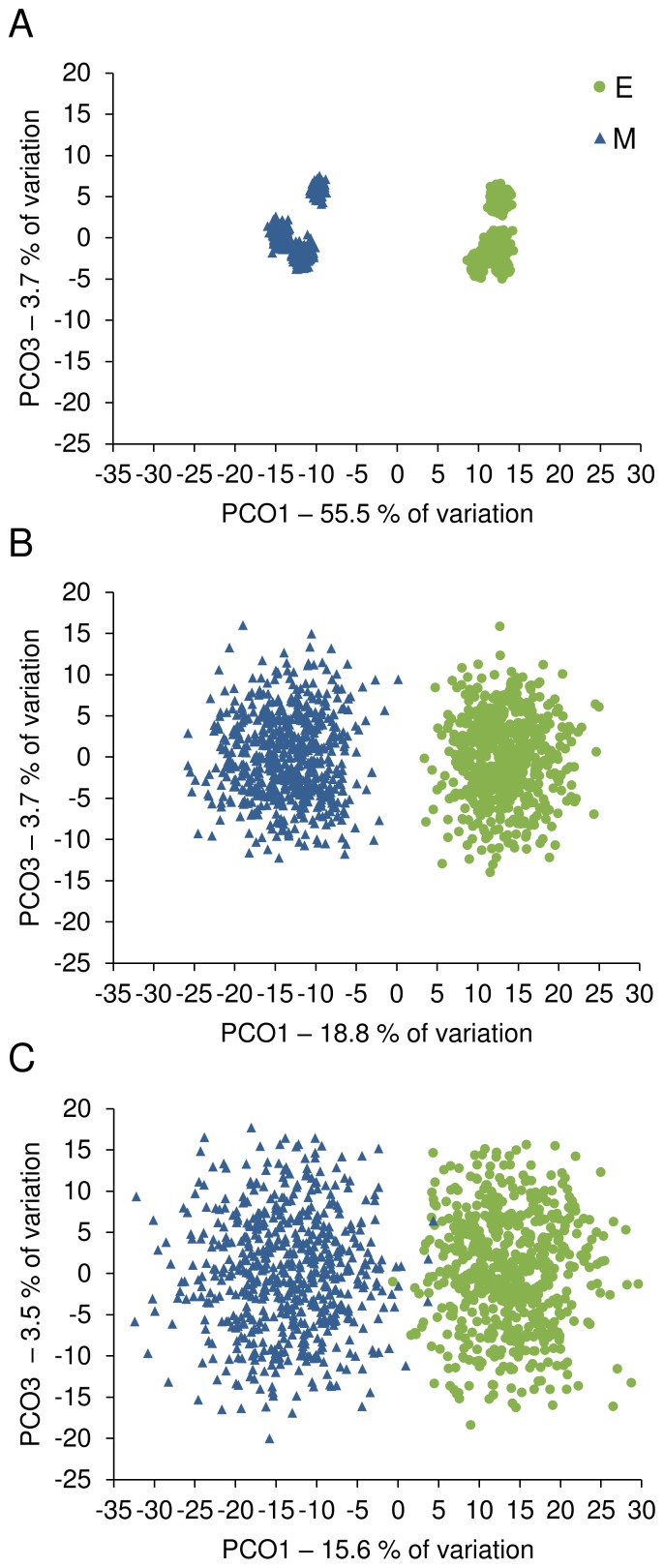
PCoA plot for the salivary bacterial communities assessed by 16S rDNA pyrosequencing. A Perl script was used for random picking of 4000 (A), 100 (B), and 50 (C) OTUs from each sample of the normalized dataset in Pipeline 6. Sub-sampling was performed 100 times and the relative abundance of OTUs obtained in each iteration was square root-transformed and used to construct the Bray-Curtis similarity matrix, which was then used in the PCoA. E, enzymatic lysis; M, mechanical lysis.

Permanova of Bray-Curtis similarities confirmed the effect of the extraction method on the structure of the salivary microbiota (Table S3). This test was based on square-root transformed abundance of OTUs and did not take into account the phylogenetic distance, as this was the case when using UniFrac. Similarly, PCoA of the Bray-Curtis similarity matrix based on square-root-transformed OTU abundance showed that samples processed using the same extraction method clustered together (Figure 4). We used this non-phylogenetic approach to analyze smaller size libraries simulated by sampling a given number of OTUs from each sample. The PCoA plots show that as few as 100 randomly chosen OTUs per sample results in a clear separation of bacterial community profiles according to the extraction method (Figure 4). Further reducing this number to 50 still showed the between-method separation, although with some overlap.

### Shared OTUs and Diversity Estimates

Pipeline 2 generated the highest total number of OTUs, as well as the highest average number of OTUs per sample (Table 1). The proportion of chimeras detected using Chimera Slayer (Pipeline 2) was lower than that obtained with UCHIME (Pipeline 1) or a BLASTN-based method (Pipeline 3) (Table 1). When the samples derived from the mechanical disruption were compared with those obtained by enzymatic lysis, the following trends were observed across all analysis pipelines: (i) lower average fraction of detected chimeras; (ii) higher average number of OTUs per sample; and (iii) greater average fraction of OTUs shared among samples. The Chao 1 richness estimator predicted a higher number of OTUs for mechanically-lysed samples in all pipelines except Pipeline 2. However, these differences exceeded the threshold of statistical significance in only a few cases. Differences in Shannon and Simpson diversity indices between the extraction methods were not statistically significant (Table 1).

## Discussion

The current study showed that the same bacterial taxa tended to be abundant and frequent in human saliva samples processed by either the mechanical or enzymatic lysis methods, but that their relative abundance, determined by pyrosequencing of 16S rDNA amplicon libraries, differed between the extraction methods. These differences could be distinguished with as few as 100 sequences per sample, i.e. far below the number of reads routinely analyzed in community studies based on HTS of 16S rDNA amplicon libraries. Similarly, Kuczynski *et al*. [35] showed that <100 sequences per sample were sufficient to discriminate between microbiota from dissimilar habitats, but underlined that such a low coverage does not allow the determination of differences in the rare biosphere.

The observed lower DNA yield, which was associated with higher relative standard deviation, in the mechanically-lysed samples points to the possibility of DNA loss during the column purification. Relatively small differences in input DNA amounts likely had very little impact on the profiling of the microbiota because the values were much higher than the 1 pg/µL threshold under which salivary microbiota profiling is significantly impacted by template DNA levels [36].

Differences in the cell wall composition and structure may explain variations in bacterial susceptibility to different lysis procedures. For instance, variation in peptide cross-links in peptidoglycan renders bacteria more or less susceptible to proteinase K lysis [37]. Several studies have shown that disruption of bacteria with tough cell walls, such as those belonging to the phyla Firmicutes and Actinobacteria, is more efficient by a mechanical approach than by an enzymatically-based protocol [36]. Our data confirmed the trend of a higher proportion of Actinobacteria in mechanically processed samples, whereas Firmicutes showed the opposite trend. However, some Firmicutes from the order Clostridiales (*Catonella*, *Eubacterium*, and *Selenomonas*) as well as a *Streptococcus* OTU had higher relative abundance when mechanical cell disruption was applied.

Hierarchical clustering of datasets based on the relative abundance of genera resulted in two main clusters corresponding to the extraction method (Figure S2). Within each of these two clusters similar trends were observed: (i) for each sample, datasets obtained using Pipelines 1–5 generally clustered together; (ii) datasets obtained for different samples using Pipeline 6, which uses reference-based OTU picking, were more similar to each other than to the datasets obtained using other pipelines. Therefore, differences between the extraction procedures outweighed the variations due to the bioinformatics analysis pipelines used.

In our study, the exact species composition/abundance of the salivary microbiota and the proportion of non-lysed bacterial cells were unknown, and therefore, it remains unclear which protocol yielded a more accurate description. Also, to determine whether differences due to the DNA extraction method outweighed inter-individual differences, as has been recently shown for bronchoalveolar lavage samples [38], it would be necessary to analyze samples from multiple individuals.

The advantage of the enzymatically-based DNA extraction method resides in its simple procedure including few steps and direct use of the lysate in PCR. This approach may be suited for samples with limited amounts of DNA template or other cases where DNA loss associated with DNA clean-up is to be avoided. However, in the absence of a DNA purification step, more PCR inhibitors may remain in the sample.

Variations in DNA extraction methods, such as the use of different cell wall-degrading enzymes, chemical agents, bead sizes, bead-beating time, and DNA purification procedures [33], [36], [38], [39] may affect profiling of the microbiota. In addition, we found that the magnitude of differences in the microbiota profiles obtained by different extraction methods is somewhat sensitive to the parameters used in bioinformatics pipelines. Therefore, caution is needed when comparing microbial community data from different studies.

## Supporting Information

Figure S1 Microbial community profiles at the class (A), order (B), family (C), and OTU (D) levels. Only taxa found at an average frequency >0.25% by at least one extraction method in at least one analysis pipeline are presented. The indicator values >0.5, determined by indicator species analysis, associated with the Benjamini-Hochberg corrected *P* values <0.05, were used to define indicators. Symbols “>” and “<” correspond to such indicator taxa and denote increasing and decreasing trends in the relative abundance for enzymatic vs. mechanical lysis. OTUs with an average abundance <0.25% for both extraction procedures (of a given analysis pipeline) were filtered out to reduce the number of comparisons in the indicator species analysis. For abbreviations, refer to Figure 1A.
(ZIP)

Figure S2 Hierarchical clustering of the 16S profiles obtained using different DNA extraction protocols and bioinformatic analysis pipelines. Group-average clustering was based on the Bray-Curtis similarity matrix computed from square-root transformed relative abundance of genera. Dataset IDs: Extraction method (E, enzymatic; M, mechanical)_Extraction # (1–3)_PCR # (1 and 2 stand for different barcode sequences in the reverse PCR primer)_Bioinformatics pipeline # (P_1–P_6). The genera *Lautropia* and *TG5*, absent in the RDP taxonomy, were excluded from the analysis.
(TIF)

Table S1 DNA yield using mechanical and enzymatic lysis protocols.
(DOCX)

Table S2 Relative abundance of taxa inferred from pyrosequenced 16S rDNA amplicon libraries. Data from six analysis pipelines are given for different taxonomic ranks (phylum, class, order, family, genus, OTU). Sample IDs: Extraction method (E, enzymatic; M, mechanical)_Extraction # (1–3)_PCR # (1 and 2 stand for different barcode sequences in the reverse PCR primer).
(XLSX)

Table S3 Differences in microbiota profiles due to the extraction procedure. Permanova test with 9,999 permutations was performed on Bray-Curtis similarity matrix based on the square root-transformed relative abundance of OTUs in six enzymatically-treated samples and six mechanically-treated samples.
(DOCX)
