# Supplementary figures and images for: Comparison of DNA Extraction Methods in Analysis of Salivary Bacterial Communities
Source: PLoS One. 2013 Jul 3;8(7):e67699. doi: 10.1371/journal.pone.0067699 (PMC3701005; doi:10.1371/journal.pone.0067699)

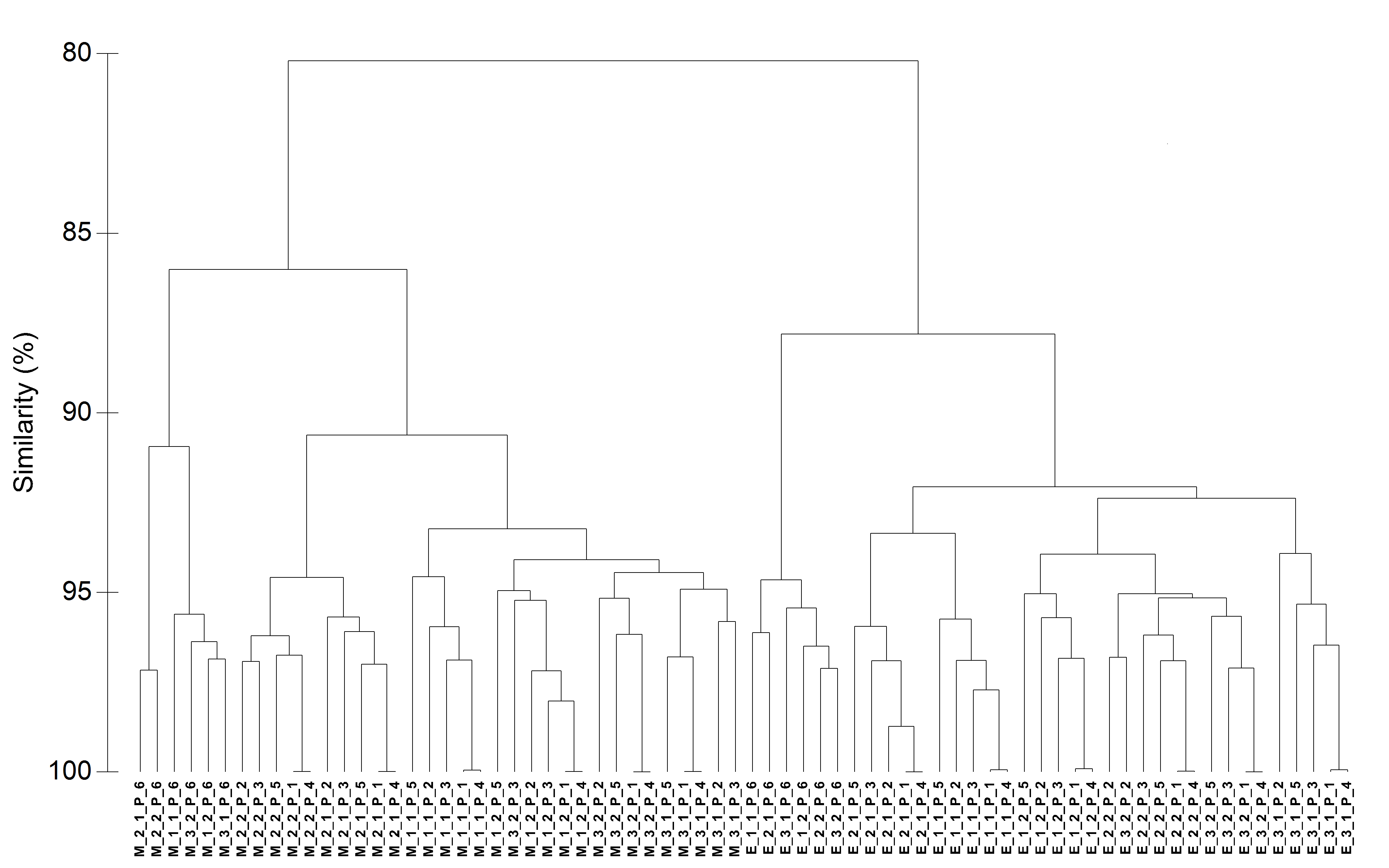

Supplement: Figure S2 — Hierarchical clustering of the 16S profiles obtained using different DNA extraction protocols and bioinformatic analysis pipelines. Group-average clustering was based on the Bray-Curtis similarity matrix computed from square-root transformed relative abundance of genera. Dataset IDs: Extraction method (E, enzymatic; M, mechanical)_Extraction # (1–3)_PCR # (1 and 2 stand for different barcode sequences in the reverse PCR primer)_Bioinformatics pipeline # (P_1–P_6). The genera Lautropia and TG5, absent in the RDP taxonomy, were excluded from the analysis. (TIF) [file pone.0067699.s002.tif]
