# Supplementary material for: Comparison of DNA Extraction Methods in Analysis of Salivary Bacterial Communities
Source: PLoS One. 2013 Jul 3;8(7):e67699. doi: 10.1371/journal.pone.0067699 (PMC3701005; doi:10.1371/journal.pone.0067699)
Supplement: Table S1 — DNA yield using mechanical and enzymatic lysis protocols. (DOCX) [file pone.0067699.s003.docx]

| **Sample code*** | **Extraction procedure** | **DNA concentration (ng/µL) in the extracts estimated by qPCR**** | | **Bacterial DNA used for the construction of the 16S amplicon library (ng)** |
| --- | --- | --- | --- | --- |
|  |  | **Human** | **Bacterial** |  |
| E_1 | enzymatic | 3.395 (6.789)*** | 0.482 (0.963) | 2.409 |
| E_2 | enzymatic | 3.679 (7.359) | 0.469 (0.938) | 2.345 |
| E_3 | enzymatic | 3.945 (7.891) | 0.451 (0.901) | 2.254 |
| M_1 | mechanical | 2.937 | 0.157 | 0.787 |
| M_2 | mechanical | 3.621 | 0.259 | 1.297 |
| M_3 | mechanical | 2.357 | 0.116 | 0.579 |

*Extraction method (E, enzymatic; M, mechanical)_Extraction # (1–3)

**Reported values are the average of duplicate (human) or triplicate (bacteria) measures. Relative deviations from the average values were <1.2%.

***Corrected values taking into account the 2-fold dilution of the saliva sample during the enzymatic extraction procedure are given in parentheses. Values for the mechanically-processed samples were not corrected because the initial saliva sample and the eluate of purified DNA had the same volume (100 µL).
