# Supplementary material for: Comparison of DNA Extraction Methods in Analysis of Salivary Bacterial Communities
Source: PLoS One. 2013 Jul 3;8(7):e67699. doi: 10.1371/journal.pone.0067699 (PMC3701005; doi:10.1371/journal.pone.0067699)
Supplement: Table S3 — Differences in microbiota profiles due to the extraction procedure. Permanova test with 9,999 permutations was performed on Bray-Curtis similarity matrix based on the square root-transformed relative abundance of OTUs in six enzymatically-treated samples and six mechanically-treated samples. (DOCX) [file pone.0067699.s005.docx]

| **Pipeline** | **Pseudo-*F*** | ***P*** |
| --- | --- | --- |
| **1** | 7.8635 | 0.0017 |
| **2** | 7.1353 | 0.0018 |
| **3** | 8.7983 | 0.0029 |
| **4** | 8.7302 | 0.0019 |
| **5** | 7.8635 | 0.0017 |
| **6** | 15.387 | 0.0029 |
